# Prevalence and Associated Factors of Suicidal Ideation among Almeda Textile Factory Workers in Adwa, Tigray, Ethiopia: A Cross-Sectional Study

**DOI:** 10.1155/2022/9459186

**Published:** 2022-12-22

**Authors:** Tesfaye Begashaw, Gebeyaw Kassie

**Affiliations:** ^1^Department of Psychiatry, College of Health Sciences, Aksum University, Aksum, Ethiopia; ^2^Department of Nursing, College of Health Sciences, Woldia University, Woldia, Ethiopia

## Abstract

**Background:**

Suicidal ideation among textile factory workers is a major public health concern worldwide and is associated with a higher risk of completed suicide. However, there are limited studies that determined the prevalence and the potential determinants of suicidal ideation in Africa, including Ethiopia. Therefore, this study is aimed at exploring the prevalence of suicidal ideation and associated factors among textile factory workers in Almeda textile factory in Adwa, Ethiopia.

**Methods:**

An institutional-based cross-sectional study design was conducted from May 3, 2020, to June 16, 2020, at Almeda textile factory in Adwa. A total of 409 workers were identified using a systematic random sampling technique. Data were collected using a self-administered questionnaire using suicidality module of Composite International Diagnostic Interview. Data were analyzed using SPSS version 22.0, and logistic regression model was employed. Adjusted odds ratio with a 95% confidence interval was computed, and statistical significance was declared at *p* value < 0.05.

**Result:**

In this study, the prevalence of suicidal ideation was found to be 8.8% (95% CI: 6.1, 11.5). Working more than 48 hours per week (AOR = 2.88, 95% CI: 1.18, 7.04), depression (AOR = 3.90, 95% CI: 1.60, 9.50), work-related physical injury (AOR = 5.95, 95% CI: 2.37, 14.94), and interpersonal conflict (AOR = 3.54, 95% CI: 1.40, 8.90) were the significant factors associated with suicidal ideation. *Conclusion and Recommendation*. In this study, suicidal ideation among Almeda textile production workers was a significant problem. Factors including depression, work-related exposure to physical injury, long working hour, and interpersonal conflict can enlarge suicidal ideation.

## 1. Introduction

Suicidal ideation (SI) commonly refers to the thought of a wish for death; it is the strong predictor of complete suicide [1]. In psychiatry, suicide is an emergency case; it causes 800, 000 people to die annually, i.e., each 40 second an individual dies by suicide some place on the globe. Suicide is a sensitive case for report because of culture, even illegal in some countries [2]; it is very likely that it is underreported. In countries with a good vital registration data, suicide may often misclassify as an accident or another cause of death, simply death uncounted [3].

In studies done in India and Vietnam, the prevalence of suicidal ideation in the textile and garment industry workers was 29.9% and 12.5% in [4, 5], respectively. In Northwest Ethiopia at Bahir Dar, 76.7% of the employee had suicidal thoughts of whom 14.9% had suicidal behavior [6]. A research finding in Bangladesh recommended that developing countries should improve their ability to prevent and treat depression to reduce incidence of suicide among industrial workers [7].

Mental health of textile industry workers and work place have a complex relationship related to job insecurity, time structure, social contact, payment, and other organizational variables. Studies showed that employee in a textile industry experienced suicidal ideation associated with a variety determinant variables such as job-related physical harm/injuries [8], job strain, long/abnormal working hours, interpersonal conflict, bullying, low salary, job unhappiness [4, 7, 9], and poor social capital [10].

Industrial workers are among the high-risk populations to suicidal ideation than the general population [11, 12]. Suicide is the second leading cause of death among ages 15-29; and the majority of industry workers are young, which makes the problem very serious. CDC reported that in the USA, suicidal rate among construction industry was 52.1% and the smallest suicide rate was recorded among education, training, and library professionals which was 5.3% [13].

The WHO sets a comprehensive multisectoral suicide prevention strategy for achieving the global target 10% by 2020 [3]. However, in Ethiopia, there is no initiative for a multisectoral suicidal prevention strategy particularly in textile industry sector. It might be because of limited evidence, even workers are in hopeless and suicidal risk situation. For instance, Ethiopian researchers showed that textile industry workers are exposed to several problems such as chronic pain (9.8%), physical harm (31.6%-49%), muscular and skeletal disorders (51.7%-53.1% and 45%), sleep disturbance, and long working hour, and majority of them hold lower level of education, and most of them are young females [14–18].

Ethiopia, on growth and transformational plan two (GTP II), set the textile sector on the front position. Currently, there are 122 textile and garment factories, and the sector employed more than two million in each year [19], of which Almeda textile factory (ALTEX) is one of the top three large textile companies in Ethiopia; hence, for the best benefit of workers' mental health and productivity of the sector, conducting a scientific study on suicidal ideation among those workers was essential. Therefore, the aim of this study was to assess the prevalence of suicidal ideation and identify associated risk factors among ALTEX production workers.

## 2. Methods and Materials

### 2.1. Study Design and Area

An institutional-based cross-sectional study was conducted from May 3, 2020, to June 16, 2020, in Adwa at Almeda textile factory. It is located in the central zone of Tigray Region, 1006 km away from the country capital city Addis Ababa. The factory was established in February 1996 E.C. The company is constructed on the 550,000 m^2^ land by including production factory, spinning factory, weaving factory, woven processing, knit production factory, knit dyeing factory, and garment factory with fully equipped mechanical and electrical workshop machines and utilities. The factory is manufacturing sustainably for more than 25 years by creating a work opportunity currently for 5696 employees (December 2019 report of human resource of ALTEX).

### 2.2. Study Subjects

All selected textile factory production workers who had been working at the study area during the data collection period and workers that had worked 6 months and above were included in the study. Workers on annual leave and on medical leave because of illness during the data collection period were excluded from the study.

### 2.3. Sample Size and Sampling Procedure

The total number of participants needed to conduct this study was calculated using a single population proportion formula with the following considerations: a standard normal distribution (*z* = 1.96) with 95% confidence interval (*α* = 0.05) and *p* = 50% (0.5), since there has been no study of the prevalence of suicidal ideation specifically in this population. The absolute precision or tolerable margin of error (*d*) was taken to be 5%. Ten percent for nonrespondents was added, bringing the total sample size to 423. Study participants from each production department were allocated proportionally. There are five production units in Almeda textile factory. These were spinning, woven processing, knitting and dyeing, garment, and weaving, and 52, 17, 15, 301, and 38 participants were selected, respectively, from each unit through proportional allocation. Simple random sampling technique (through a computer-generated randomization method) was used to pick workers from each unit.

### 2.4. Operational Definitions

*Suicidal ideation*: if the respondent answers “yes” to the question “have you seriously thought about committing suicide at least ones in your life” based on the module of CIDI [20]

*Social support*: based on Oslo-3 social support scale which ranges from 3 to 14, those respondents who was scored 3–8 are considered as having poor social support, scored 9–11 are considered as having moderate social support, and scored 12-14 are considered as having strong social support [21]

*Depression*: based on PHQ-9 which ranges 0-27, individuals who scored ≥10 are considered as having depression [22]

*Chronic medical illness*: when subjects have proven medical or surgical illness such as diabetes, HTN, heart disease, HIV, epilepsy, asthma, and cancer

*Current substance use*: those who use the substance (alcohol, khat, cigarettes, and others) for nonmedical purposes in the last 3 months

*Ever substance use*: those who use the substance (alcohol, chat, cigarettes, and others) for nonmedical purposes in their lifetime

*Textile production workers*: workers who are working in textile factory to prepare natural tic fibers for spinning into yarn and yarn into textile products and combining, spinning, weaving, knitting, or bonding yarns and textiles and dyeing and finishing step up to household goods [18]

*Occupational injury*: any physical injury condition sustained by a worker in connection with the performance of his or her work in textile factories [23]

### 2.5. Data Collection Tools

The data were collected using a structured and semistructured self-administered questionnaire having components of sociodemographic characteristics, clinical factors, and work-related factors. Depression was assessed by PHQ-9 (PHQ has a total score of 27; participants who scored 10 and above are considered as having depression and those who scored less than 10 are considered as no depression) [22]. Social support was collected by the Oslo-3 item social support scale; it has 3 item questions (according to Oslo-3 social support scale which ranges from 3 to 14, those respondents who scored 3–8 are considered as having poor social support, scored 9–11 are considered as having moderate social support, and scored 12-14 are considered as having strong social support). Data on the main variable (suicidal ideation) was assessed by using CIDI; its Amharic version is validated both in clinical and community settings in Ethiopia [20].

### 2.6. Data Quality Assurance

A standardized questionnaire was prepared in English and then translated to Tigrigna and translated back to English to check for consistency and understandability of the tool. A pretested questionnaire was used to collect information. A pretest was done two months before the actual data collection with 5% [22] of the sample size at Apparel Velocity Company (Mekelle city). During data collection, a filled questionnaire was checked for completeness and consistency. Data was collected by 4 psychiatric professionals and supervised by other 2 master's mental health professionals, and they were trained about data collection. Continuous follow-up and supervision was done by the supervisors and the principal investigator.

### 2.7. Data Analysis Procedure

Data was entered and cleaned by using EpiData manager version 4.6 and exported to statistics package for Social Science version 22.0 for statistical analysis. Descriptive statistics were computed through percentage, frequency, standard deviation, and mean and presented in tables.

Binary logistic regression was used to determine the statistical association between a set of independent variables and the dependent variable. Variables with *p* value < 0.2 in the bivariate analysis were included in the multivariate analysis. The Hosmer-Lemeshow goodness of model fitness was checked, and *p* values less than 0.05 were considered statistically significant, and point estimates were presented using their respective 95% confidence intervals.

Of the total calculated 423 patients, a total of 409 participants were accessed and included in the analysis (14 patients were refused to participate in the study).

## 3. Results

### 3.1. Sociodemographic, Psychosocial Factors, and Work-Related Characteristics of the Respondents

The response rate was 96.7% (409). The mean age of the workers was 28.48 with SD ± 7.47, and it ranges from 18 to 54 years. Majority of the participants were females 260 (63.6%), and all of them were Tegaru in ethnicity. Majority of the study participants were orthodox in religion (401, 98%) and the remaining eight (2%) were Muslims.the remaining eight (2%) were muslim religion followers. More than half were single (227, 55.50%), and around 49.6% of the participants had diploma level of education. One hundred eighty-five (64.1%) participants' monthly salary was less than or equal to 2500 ETB. Of the respondents, one hundred eighty-nine (46.2%) reported a poor social support, and one hundred eighty-six (45.5%) of the participants had moderate social support, whereas thirty-four (8.3%) reported that they were getting good social support. One hundred ninety-seven (48.2%) of the respondents had from six months to five years of work experience, and one hundred fifty-seven (38.4%) were working for more than 48 hours per week. One hundred seventy-seven (43.3%) were working by rotation (day and night) and 231 (56.5%) working fixed night time. Majority of the workers (89.5%) had no additional jobs, and 161 (39.4%) were loaded for work. Two hundred eighty-nine (70.7%) of the respondents were working under garmenting department. One hundred ten (26.9%) and 93 (22.7%) of the respondents experienced interpersonal conflict and injuries related to work, respectively. One hundred thirty-five (67.5%) of the respondents' were not satisfied with their job (Table 1).

### 3.2. Clinical and Substance Use Characteristics

Almost half (51.3%) of the participants had drunk alcohol in their lifetime and 47.9% drunk in the last three months. On the clinical aspect, 10.3% of participants had medical history (AIDS, DM, HPT, and others, 0.7%, 1.5%, 3.2%, and 4.9%, respectively) and 3.2% had a family history of suicide. Eighty-seven (21.3%) of respondents had depression (Table 2).

### 3.3. Prevalence of Suicidal Ideation

The lifetime prevalence of suicidal ideation was 8.8% (36) (95% CI: 6.1, 11.5), of whom 2.4% [10] had thought commuting suicide within the last one month and 2.9% [12] had a plan for commuting suicide.

### 3.4. Factors Associated with Suicidal Ideation among Almeda Textile Factory Production Workers

In the bivariate logistic regression analysis, variables being female, marital status, educational status, poor social support, interpersonal conflict at work place, work load, long working hours, injury at work place, job satisfaction, medical history, past psychiatric history, and depression were found to be significant predictors of suicidal ideation among participants with a *p* value of less than 0.2.

After the above crude analysis, multivariable logistic regression was done for predicting suicidal ideation among participants adjusted for all possible candidate predictors pooled from bivariate logistic regression analysis, and interpersonal conflict at work, long working hours, injuries at work place, and depression were found to be statistically significant predictors of suicidal ideation with *p* value < 0.05.

Respondents who were working for more than 48 hours per week had a suicidal ideation of nearly three times higher than those who had been working less than or equal to 48 hours per week. Participants who had conflict at the work place had a suicidal ideation of 3.54 times higher than those who had no suicidal ideation. Workers who experienced work injury were six times more likely to have suicidal ideation compared with those who did not experience work injuries. Participants with depression were also 4 times higher to have suicidal ideation when compared to patients who had no depression (Table 3).

## 4. Discussion

This study determined the magnitude of suicidal ideation and identified the factors associated with this major problem through self-administered questions of 409 selected ALTEX workers.

In this study, the prevalence of suicidal ideation was found to be 8.8% (95% CI: 6.1-11.8). This is lower than the study carried out in Vietnam (12.5%) [5] and in India (29.9%) [4]. The possible reasons of variation in Vietnam might be the cultural variability and the measurement tool they used; CIDI was used in this study, whereas a general health questionnaire (PHQ) was used both in Vietnam and India. Other reasons might be, in the Indian study only female study participants with age group of 12-28 years were included; it is known that these age groups and females are more exposed to suicidal thoughts.

Regarding factors, participants who had depression were more likely to have suicidal ideation than participants who had no depression, which means 58.33% of those who had depression had suicidal ideation. This might be justified by the fact that people with mental illness are high risk for suicide with 3 to 12 times and 80% of depressive patients have risk for suicidal behavior [1]. This is also supported by a study done in Japan; 70% of employees who had suicidal ideation were depressed [24].

Participants who were working more than 48 hours a week had a significant association with suicidal ideation when compared to those who were working less than or equal to 48 hours a week; in this study, 157 (38.5%) of the study participants work more than 48 hours a week, and 24 (15.7%) of them have suicidal thoughts. Other studies showed that working long hours and evening and night shift working were associated with suicidal ideation [8, 24, 25]. This is also supported by studies done in Austria, Japan, and Korea [26–28].

In this study finding, participants who had faced work place physical injuries were more likely to have suicidal ideation than those who had not faced work place physical injuries. This is supported by prior studies; workers experiencing physical injury can experience hopelessness and wish to death [8]. Another study done in Korea showed that work place injury makes workers to feel bad and decreased their quality of life [27]. The World Health Organization also reported that exposure to injuries leads to suicidal behavior [3].

Workplace interpersonal conflict was another factor significantly associated with suicidal ideation. Those workers who faced work place interpersonal conflicts were more likely to have suicidal ideation as compared to those who were not facing work place interpersonal conflicts. If employees are exposed to unnecessary arguments and conflicts at their workplace, they may become discouraged and have suicidal thoughts.If employees are exposed to conflict at work, it might be easily lead them to suicidal thoughts [29]. This is also supported by a study done in Japan, if there was low workplace social capital statistically associated with suicidal ideation [10]. Similar study done in Korea also showed that one of the predominant causes of suicidal ideation among employees was interpersonal conflict [27, 30].

When we conclude the study, suicidal ideation in Almeda textile factory workers is an important public mental health problem. We took an adequate sample size as strength and used cross-sectional study design as a weakness that is difficult to show cause and effect relationship.

## Figures and Tables

**Table 1:** Distribution of participants by sociodemographic, psychosocial support, and work-related factors of Almeda textile factory production workers in Adwa, Tigray, Ethiopia, 2020 (*N* = 409).

Variable	*N*	%
Age		
18-27	209	51.1
28-37	141	34.5
>37	59	14.4
Sex		
Female	260	63.6
Male	149	36.4
Marital status		
Single	227	55.5
Divorced/widowed	19	4.6
Married	163	39.9
Religion		
Orthodox	401	98
Muslim	8	2
Educational status		
Elementary	62	15.2
Secondary	100	24.4
Diploma	203	49.6
Degree	44	10.8
Residency of family		
Rural	54	13.2
Urban	355	86.8
Salary		
≤2500	262	64.1
2501-3500	44	10.8
3501-5000	57	13.9
>5000	46	11.2
Psychosocial support		
Poor	189	46.2
Moderate	186	45.5
Strong	34	8.3
Working experience		
≤5 years	197	48.2
>5 years	212	51.8
Working hours a week		
>48 hr	157	38.4
≤48 hr	252	61.6
Working shift		
Rotation (day and night)	177	43.5
Fixed	231	56.5
Second job		
Yes	43	10.5
No	366	89.5
Work load		
Yes	161	39.4
No	248	60.6
Department		
Garmenting	289	70.7
Spinning	51	12.5
Knitting and knit dyeing	17	4.2
Weaving	36	8.8
Woven processing	16	3.8
Interpersonal conflict		
Yes	110	26.9
No	299	73.1
Participants' physical injury		
Yes	93	22.7
No	316	77.3
Job satisfaction		
No	276	67.5
Yes	133	32.5

*N* = number; % = percent.

**Table 2:** Description of clinical and substance-related factors among Almeda textile factory production workers in Adwa, Central Tigray, Northern Ethiopia, 2020 (*N* = 409).

Variables	*N*	%
Family history of suicide		
Yes	13	3.2
No	396	96.8
Past psychiatry history		
Yes	5	1.2
No	404	98.9
Depression		
Yes	87	21.3
No	322	78.7
Medical history		
Yes	42	10.3
No	367	89.7
Comorbid medical illness		
AIDS	3	0.7
DM	6	1.5
HPT	13	3.2
Others^∗^	20	4.9
Any alcoholic use history in the last 3 months		
Yes	196	47.9
No	213	52.1
Ever use of alcohol		
Yes	210	51.3
No	199	48.7
Any smocking use history in the last 3 months		
Yes	21	5.1
No	388	94.9
Ever tobacco use		
Yes	23	5.6
No	386	94.4
Other substance use history in the last 3 months^∗∗^		
Yes	14	3.4
No	395	96.6
Ever use of other substance		
Yes	17	4.2
No	392	95.8

Others ^∗^ = epilepsy and cardiac-related problems; ^∗∗^ = cannabis and hashish; *N* = number; % = percent.

**Table 3:** Association between factors that associated with bivariate logistic regression (*p* value < 0.25) and suicidal ideation among Almeda textile factory production workers in Adwa, Tigray, Ethiopia, 2020 (*N* = 409).

Variables	SI		AOR	*p* value
	Yes	No		
Sex				
Female	28	232	1.36 (0.48, 3.80)	0.56
Male	8	141	1	
Marital status				
Single	24	203	2.17 (0.88, 5.93)	0.13
Divorced/widowed	4	15	3.45 (0.73, 16.43)	0.12
Married	8	155	1	
Educational status				
Elementary	2	60	1.14 (0.07, 17.68)	0.82
Secondary	9	91	3.97 (0.35, 45.40)	0.27
Diploma	24	179	3.56 (0.35, 36.25)	0.28
Degree	1	43	1	
Working hour				
>48 hr	24	133	2.88 (1.18,7.04)	0.02
≤48 hr	12	240	1	
Workload				
Yes	25	136	1.11 (0.43, 2.88)	0.83
No	11	237	1	
Conflict at work				
Yes	24	86	3.54 (1.40, 8.90)	0.01
No	12	287	1	
Injury at work place				
Yes	24	69	5.95 (2.37, 14.94)	0.00^∗^
No	12	304	1	
Job satisfaction				
No	28	248	0.62 (0.22, 1.74)	0.36
Yes	8	125	1	
Depression				
Yes	21	66	3.90 (1.60, 9.50)	0.00^∗^
No	15	307	1	
Psychiatric history				
Yes	2	3	10.29 (0.85, 124.37)	0.07
No	34	370	1	
Medical history				
Yes	8	34	1.40 (0.41, 4.75)	0.59
No	28	339	1	

AOR = adjusted odds ratio; SI = suicidal ideation; 1 = reference; ^∗^ = *p* value < 0.01.

## Data Availability

All raw data included in the manuscript can be accessed from the corresponding author through the email address of Gebeyaw Molla (mollagebeyaw@gmail.com) with rational request.

## Abbreviations

AOR: Adjusted odds ratio
ALTEX: Almeda textile factory
CI: Confidence interval
CIDI: Composite International Diagnostic Interview
COR: Crude odds ratio
SI: Suicidal ideation
WHO: World Health Organization.
